# Neuron Class and Target Variability in the Three-Dimensional Localization of SK2 Channels in Hippocampal Neurons as Detected by Immunogold FIB-SEM

**DOI:** 10.3389/fnana.2021.781314

**Published:** 2021-12-15

**Authors:** Rafael Luján, Angel Merchán-Pérez, Joaquim Soriano, Alejandro Martín-Belmonte, Carolina Aguado, Rocío Alfaro-Ruiz, Ana Esther Moreno-Martínez, Javier DeFelipe

**Affiliations:** ^1^Synaptic Structure Laboratory, Instituto de Investigación en Discapacidades Neurológicas (IDINE), Departamento de Ciencias Médicas, Facultad de Medicina, Universidad Castilla-La Mancha, Albacete, Spain; ^2^Laboratorio Cajal de Circuitos Corticales, Centro de Tecnología Biomédica, Universidad Politécnica de Madrid, Madrid, Spain; ^3^CRIB-Facultad de Medicina, Universidad Castilla-La Mancha, Albacete, Spain; ^4^Instituto Cajal (CSIC), Madrid, Spain

**Keywords:** SK channels, spines, dendritic shafts, 3D-reconstruction, synapses, electron microscopy, FIB/SEM

## Abstract

Small-conductance calcium-activated potassium (SK) channels are crucial for learning and memory. However, many aspects of their spatial organization in neurons are still unknown. In this study, we have taken a novel approach to answering these questions combining a pre-embedding immunogold labeling with an automated dual-beam electron microscope that integrates focused ion beam milling and scanning electron microscopy (FIB/SEM) to gather 3D map ultrastructural and biomolecular information simultaneously. Using this new approach, we evaluated the number and variability in the density of extrasynaptic SK2 channels in 3D reconstructions from six dendritic segments of excitatory neurons and six inhibitory neurons present in the *stratum radiatum* of the CA1 region of the mouse. SK2 immunoparticles were observed throughout the surface of hippocampal neurons, either scattered or clustered, as well as at intracellular sites. Quantitative volumetric evaluations revealed that the extrasynaptic SK2 channel density in spines was seven times higher than in dendritic shafts and thirty-five times higher than in interneurons. Spines showed a heterogeneous population of SK2 expression, some spines having a high SK2 content, others having a low content and others lacking SK2 channels. SK2 immunonegative spines were significantly smaller than those immunopositive. These results show that SK2 channel density differs between excitatory and inhibitory neurons and demonstrates a large variability in the density of SK2 channels in spines. Furthermore, we demonstrated that SK2 expression was associated with excitatory synapses, but not with inhibitory synapses in CA1 pyramidal cells. Consequently, regulation of excitability and synaptic plasticity by SK2 channels is expected to be neuron class- and target-specific. These data show that immunogold FIB/SEM represent a new powerful EM tool to correlate structure and function of ion channels with nanoscale resolution.

## Introduction

CA1 pyramidal neurons are involved in key neuronal circuits that underlie cognition, memory and anxiety (Spruston, 2008), and they are increasingly recognized as vulnerable cell types that contribute to the pathogenesis of epilepsy and Alzheimer’s disease (Anderson et al., 2007; Small et al., 2011; Merino-Serrais et al., 2013). One of the key regulators of CA1 neuronal activity is the small-conductance Ca^2+^-activated K^+^ (SK; KCNN) channels. SK channels are voltage-independent, directly gated by submicromolar concentrations of cytosolic Ca^2+^ and blocked by apamin (Köhler et al., 1996; Ngo-Anh et al., 2005; Luján et al., 2009). To date, four members of the mammalian SK gene family (KCNN1/KCa2.1/SK1, KCNN2/KCa2.2/SK2, KCNN3/KCa2.3/SK3, and KCNN4/KCa3.1/SK4) have been identified, although only SK1, SK2 and SK3 are expressed in the rodent hippocampus (Köhler et al., 1996; Stocker and Pedarzani, 2000; Sailer et al., 2004).

The SK2 subunit emerged as a prominent player in the hippocampus, where it accounts for most physiological roles of SK channels in CA1 pyramidal neurons. Previously, it has been shown that activation of SK2 channels modulates synaptic responses, plasticity, memory encoding, and contributes to the expression and maintenance of long-term potentiation (LTP) (Bond et al., 2004; Hammond et al., 2006; Lin et al., 2008, 2010). Furthermore, SK2-containing channels were found to be neuroprotective in ischemia-induced neuronal cell death (Allen et al., 2011b), as well as in drug-induced plasticity (Fakira et al., 2014). In relation to glutamate excitotoxicity, SK2 channel activation blocks pathological Ca^2+^ influx from the extracellular space, thus promoting neuroprotection (Dolga and Culmsee, 2012). Taken together, all these functions for SK2 channels reflect their localization to dendritic spines (for simplicity, spines), where they couple with different Ca^2+^ sources, mainly NMDA and mGlu_5_ receptors, in distinct membrane domains (Lin et al., 2008; Ballesteros-Merino et al., 2012; García-Negredo et al., 2014). Despite the progress on SK channel physiology and physiopathology (Adelman et al., 2012; Kshatri et al., 2018; Sun et al., 2020), data on the spatial organization of SK channels in neurons is still scarce. The approach to decipher the molecular organization at the ultrastructural level is immunoelectron microscopy.

In the present study, we report a novel approach for 3D mapping of SK2 channels by combining the use of pre-embedding immunogold labeling method, with volume electron microscopy, performed by focused ion beam milling and scanning electron microscopy (FIB-SEM). Specifically, we studied several aspects on the organization of 4775 immunoparticles for SK2 in six reconstructed dendrites of pyramidal cells that were identified by the presence of dendritic spines, and 1574 immunoparticles for SK2 in six reconstructed dendrites of inhibitory neurons. In particular, we analyzed the density of SK2 in dendritic spines and shafts of pyramidal neurons, and the density of SK2 inmmunoparticles in dendritic shafts devoid of spines, belonging to inhibitory neurons. We also quantified the ratio of plasma membrane vs. intracellular distribution of SK2, the 3D spatial distribution of SK2 clusters on the surface, and the spatial location of SK2 relative to asymmetric and symmetric synapses, which generally correspond to excitatory glutamatergic synapses and GABAergic inhibitory synapses, respectively (e.g., Ascoli et al., 2008). This work is the first detailed report of the 3D molecular organization of SK2 channels in the *stratum radiatum* of the rodent hippocampal CA1 field with a level of detail never previously attained. Our approach offers a new way to investigate the molecular organization of ion channels contributing to the analysis of hippocampal circuits in their native environment.

## Materials and Methods

### Animals and Tissue Preparation

Three C57BL/6 mice five-week-old (Charles River Laboratories, Barcelona, Spain) housed in the Animal House Facilities of the Universidad de Castilla-La Mancha (Albacete, Spain) were used in the study. Care and handling of animals prior to and during experimental procedures was in accordance with European Union regulations (86/609/EC), and the protocols were approved and supervised by the local Animal Care and Use Committee.

Animals were deeply anesthetized by intraperitoneal injection of ketamine-xylazine 1:1 (ketamine, 100 mg/Kg; xylazine, 10 mg/Kg). Once reflex activity was completely abolished, the heart was surgically exposed for perfusion fixation through the ascending aorta, first with 0.9% saline and then followed by freshly prepared ice-cold fixative containing 4% paraformaldehyde, 0.05% glutaraldehyde, and ∼0.2% picric acid, made up in 0.1 M phosphate buffer (PB; pH 7.4). After perfusion, brains were removed and immersed in the same fixative for 2 h or overnight at 4°C. Tissue blocks were washed thoroughly in 0.1 M PB. Coronal 60 μm thick sections were cut on a Vibratome (Leica V1000).

### Antibodies and Chemicals

An affinity-purified polyclonal antibody against SK2 was used which was raised in guinea pig (GP-Af540; aa. 536–574 of mouse SK2; RRID:AB_2571841; Frontier Institute Co., Japan) and characterized previously (Cueni et al., 2008; Lin et al., 2008). The secondary antibody used was goat anti-guinea pig IgG coupled to 1.4 nm gold (1:100; Nanoprobes Inc., Stony Brook, NY, United States).

### Pre-embedding Immunogold for Electron Microscopy

Immunohistochemical reactions for electron microscopy were carried out using the pre-embedding immunogold method as described previously (Luján et al., 1996). Briefly, free-floating vibratome sections were incubated in 10% (v / v) NGS diluted in tris buffered saline (TBS). Sections were then incubated in anti-SK2 [3–5 μg / mL diluted in TBS containing 1% (v / v) NGS], followed by incubation in goat anti-guinea pig IgG coupled to 1.4 nm gold (Nanoprobes Inc., Stony Brook, NY, United States). Sections were postfixed in 1% (v / v) glutaraldehyde and washed in double-distilled water, followed by silver enhancement of the gold particles with an HQ Silver kit (Nanoprobes Inc.). Sections were then treated with osmium tetraoxide (1% in PB), block-stained with uranyl acetate, dehydrated in graded series of ethanol and flat-embedded on glass slides in Durcupan (Sigma-Aldrich, St. Louis, MO, United States) resin. Hippocampal regions of interest (*stratum radiatum* of the CA1 field) were cut at 70–90 nm on an ultramicrotome (Reichert Ultracut E, Leica, Vienna, Austria) and collected on single slot pioloform-coated copper grids.

### Dual Been Electron Microscopy (FIB/SEM)

Selected areas of the CA1 region from embedded vibratome sections were glued onto a blank Durcupan block and trimmed. We focused on the neuropil of the *stratum radiatum* that only contained axons, dendrites, and glial processes. To select the exact location to be imaged, ultrathin sections were only collected close to the surface of each block, as immunoreactivity decreases with depth with the pre-embedding immunogold technique (Luján et al., 1996). For that purpose, it is important to make ultrathin sectioning cuts that are not perpendicular to the longitudinal axis of tissue surface. Thus, tissue block should be cut tilted in a proper angle so that the ultrathin sections contain an area with tissue, one area with only resin and the interface between resin and tissue among both areas. Then, we selected areas of the *stratum radiatum* with optimal gold labeling at approximately the same distance from the interface resin/tissue, which was defined within 20 to 30 μm from the interface. We estimated the quality of immunolabeling using a JEOL JEM-1011 (JEOL Ltd., Tokyo, Japan) transmission electron microscope equipped with a digitalizing image system (SC1000 ORIUS, 11 megapixels; Gatan, Pleasanton, CA, United States). The resin blocks containing the embedded sample were glued onto aluminum sample stubs using conductive carbon adhesive tabs (Electron Microscopy Sciences, Hatfield, PA). To prevent charging artifacts, all the surfaces of the Durcupan blocks were covered with colloidal silver paint (Electron Microscopy Sciences, Hatfield, PA), with the only exception for the top surface containing the tissue to be studied. The stubs with the mounted blocks were then placed into a sputter coater (Emitech K575X, Quorum Emitech, Ashford, Kent, United Kingdom) and were coated with gold/palladium for 15 to 30 s to facilitate charge dissipation.

A FIB/SEM equipment was used for the ultrastructural 3D study of the *stratum radiatum* (Crossbeam^®^ Neon40 EsB, Carl Zeiss NTS GmbH, Oberkochen, Germany), as described previously (Merchán-Pérez et al., 2009). This instrument combines a high-resolution field-emission SEM column (Gemini^®^ column, Carl Zeiss NTS GmbH, Oberkochen, Germany) with a focused gallium ion beam (FIB), which permits thin layers of material to be removed from the sample surface on a nanometer scale. After removing (milling) one layer of material by the FIB, the freshly exposed surface of the sample is imaged by the SEM using the backscattered (EsB) electron detector. The milling and imaging processes were sequentially repeated, and long series of images were acquired through a fully automated procedure, thus obtaining a stack of images that represented a 3D sample of the tissue (Merchán-Pérez et al., 2009).

Each photomicrograph size was 2048 × 1536 pixels and covered a field of view of 10.24 × 7.68 μm, resulting in an image resolution in the xy plane of 5 nm/pixel. Resolution in the z-axis was 20 nm, corresponding to the layer of material milled by the FIB in each cycle (section thickness), which was the same in all samples. The number of serial sections obtained for each sample (2 stacks of images were taken per animal) varied between 180 and 270. The total tissue volume that was imaged by FIB/SEM microscopy was a minimum 283.12 μm^3^ and a maximum of 424.67 μm^3^. The milling current of the FIB was 700 pA and the SEM was set to 1.7–1.8 kV acceleration potential.

### Alignment and Reconstruction of Dendrites in the Stacks of Serial Sections

For the alignment (registration) of the stack of images we used Fiji software (Schindelin et al., 2012^1^), applying a rigid registration method (translation only, no rotation) to avoid any deformation of single sections. After registration, the resulting stack was filtered and cropped. We used a Gaussian Blur filter with Fiji to eliminate noisy pixels. Then, an improved version of the software package Reconstruct (Fiala, 2005) was used to trace and carry out the serial 3D reconstruction of the labeled dendritic shafts, spines, and synaptic contacts. Each structure was assigned a different name and color, and it was manually traced through the stacks of images. Once segmentation of these structures was performed, the generated meshes were exported to vrml format. Dendrites were chosen for 3D analysis based on (1) containing immunoparticles for SK2 on plasma membrane and cytoplasmic sites, and (2) spanning at least 80 consecutive serial sections.

### Quantitative Analysis

To establish the relative abundance of SK2 immunoreactivity in the plasma membrane and intracellularly, we counted immunoparticles identified in the reconstructed dendrites of CA1 excitatory and inhibitory neurons. We reconstructed 6 dendritic segments for CA1 excitatory neurons and 6 for inhibitory neurons (dendrites identified as 1 and 2 came from one mouse, dendrites 3 and 4 came from a second mouse, and dendrites 5 and 6 came from a third mouse). Plasma membrane-bound immunoparticles were considered as such when they contacted the inner leaflet of lipid bilayer, whereas immunoparticles not in contact with the plasma membrane were considered associated to intracellular sites. The data were expressed as a percentage of immunoparticles for SK2 in each subcellular compartment in the two neuron classes (Table 1).

**TABLE 1:** Distribution of SK2 immunoparticles on 3D reconstructed dendritic spines and shafts of CA1 pyramidal cells. PM, Plasma membrane; Intra, intracellular.

	Dendritic shafts			Spines		
	Gold PM	Gold Intra	Total	Gold PM	Gold Intra	Total
Dendrite 1	444 (36,3%)	778 (63,7%)	1222	98 (96,1%)	4 (3,9%)	102
Dendrite 2	353 (61,9%)	217 (38,1%)	570	49 (90,7%)	5 (9,3%)	54
Dendrite 3	167 (23,3%)	550 (76,7%)	717	118 (98,3%)	2 (1,7%)	120
Dendrite 4	48 (3,9%)	1156 (96,1%)	1204	14 (93,3%)	1 (6,7%)	15
Dendrite 5	48 (36,4%)	84 (63,6%)	132	14 (93,3%)	1 (6,7%)	15
Dendrite 6	152 (29,5%)	363 (70,5%)	515	105 (96,3%)	4 (3,7%)	109
**Total**	**1212 (27,8%)**	**3148 (72,2%)**	**4360**	**398 (95,9%)**	**17 (4,1%)**	**415**

Although most inhibitory neurons in the hippocampus have smooth dendrites, a small number of them also contain some spines (Scheuss and Bonhoeffer, 2014). Double labeling in pre-embedding would allow us to differentiate between spine originating from excitatory or inhibitory neurons, but it decreases the efficiency of immunogold labeling, which sequentially is done after HRP reaction. Therefore, identification of spiny inhibitory neurons was only based on ultrastructural criteria. Spines of inhibitory neurons had on average longer necks (Stepanyants et al., 2002). Using this criteria, spines used for the 3D quantitative analysis were on average similar among them, making unlikely the mixing of spines originated from different neuron classes.

Clustering analysis was performed by means of a nearest neighbors’ distance (NND) approach, i.e., each particle distance to its closest neighbor was measured and its average and standard deviation computed. Particles that were closer than the average NND and two times its standard deviation were considered to belong to the same cluster. As we were interested in cluster analysis of particles embedded in the plasma membrane, particle distances were computed following its surface, rather than considering the Euclidean distances. To assess whether the overall particles were clustered or randomly distributed, we repeated the NND analysis on a hundred self-built control images (i.e., multiple rounds of random distributing particles in the same images). These images shared the same plasma membranes as the original ones and the same number of particles, which were randomly distributed. We assessed the probability of the actual NND mean occurring by chance by comparing it to the random NND means distribution. Finally, each SK2 particle minimum distance to the closest excitatory or inhibitory synapse following the plasma membrane was also computed. Both NND and closest synapse analysis were automated by means of an ImageJ (Schneider et al., 2012) script on FIJI (Schindelin et al., 2012) that implemented MorphoLibJ methods; since some of these methods assume bicubic pixels, images were resized in x-y plane to a final pixel size of 20 nm (Legland et al., 2016).

### Controls

To test method specificity in the procedures, the primary antibody was either omitted or replaced with 5% (v/v) normal serum of the species of the primary antibody, resulting in the total loss of the signal.

### Data Analysis

Statistical analyses for morphological data were performed using GraphPad Prism (San Diego, CA, United States) and data were presented as mean ± SEM. Statistical significance was defined as *P* < 0.05. The statistical evaluation of the immunogold densities was performed using the one-way ANOVA test and Bonferroni *post hoc* test. The statistical evaluation of the spine volumes was performed using the Mann Whitney test because the distribution of these data were not normal. For the electron microscopic data, statistical significance in the distribution of gold particles among samples was assessed with the Kolmogorov–Smirnov non-parametric test. The statistical evaluation of particles clustering over the plasma membrane surface was performed by means of a z score, computing the probability of the obtained NNDs’ particles distribution belonging to the randomly distributed particles population.

## Results

### Study of SK2-Immunopositive Dendrites by FIB/SEM

**FIGURE 1:**
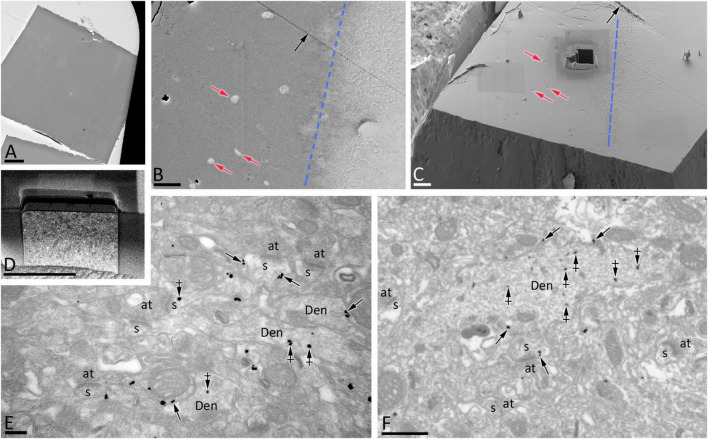
FIB/SEM microscopy combined with pre-embedding immunogold allows high-resolution 3D mapping of SK2 in neurons. **(A)** TEM ultrathin image of the resin block surface allows the visualization of tissue-resin interface to select optimal areas of SK2 immunolabelling. **(B,C)** SEM image of the block surface, revealing conserved landmarks (red arrows for blood vessels, black arrow for a knife mark), allows the selection of the starting plane for serial image acquisition close to the tissue-resin interface (blue line). A trench has been milled close to the tissue-resin interface (blue line) to gain access to the region of interest. **(D)** Low magnification SEM backscattered electron image showing a milled surface of the trench face during one of the milling-imaging cycles. **(E)** TEM ultrathin image showing immunolabelling for SK2 in the plasma membrane (arrows) and intracellular sites (crossed arrows) of spines (s) and shafts (Den) in the *stratum radiatum*. Several axon terminals (at) are also visible. **(F)** FIB/SEM image of the same area and block to that illustrated in panel **(E)** showing SK2 immunolabelling in the plasma membrane (arrows) and intracellular sites (crossed arrows) of spines (s) and shafts (Den). Some axon terminals have also been labeled (at). Scale bars: **(A)** 100 μm; **(B–D)** 20 μm; **(E)** 200 nm; **(F)** 1 μm.

**FIGURE 2:**
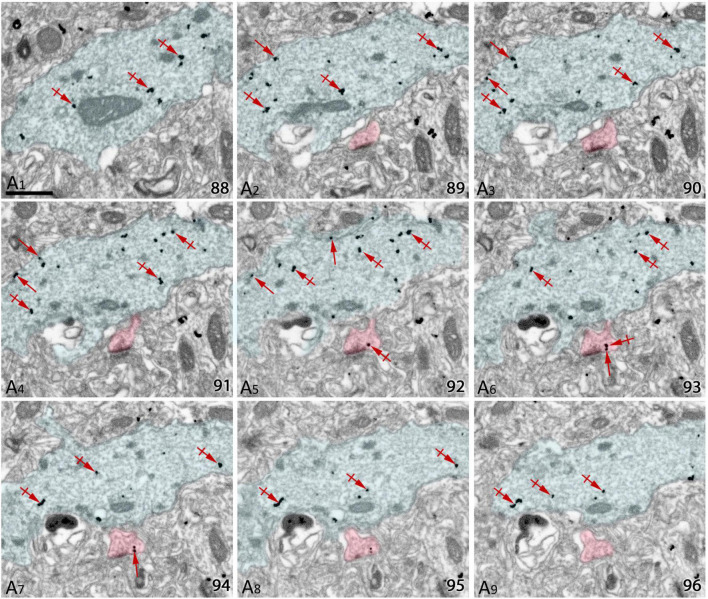
Serial images obtained by FIB/SEM from the neuropil of hippocampal CA1. **(A1–A9)** Selected sections (88–96) from a FIB/SEM stack of images obtained from the *stratum radiatum* of the CA1 field illustrating dendritic shafts (pseudo colored in blue) and spines (pseudo colored in red) of pyramidal cells immunolabelled for SK2. Immunoparticles for SK2 can be visualized along the plasma membrane (arrows) and at cytoplasmic sites (crossed arrows). Scale bars: **(A)** 1 μm.

To establish quantitative 3D anatomical reconstructions of ion channel distributions, hippocampal slices were labeled with antibodies against SK2, processed for pre-embedding immunogold, and plastic embedded using conventional TEM procedures. Ultrathin sectioning of slices and their visualization using TEM, allowed us to identify the optimal labeling for SK2, close to the interface resin-tissue, and the subsequent correlation procedure using landmarks to apply FIB/SEM technology (Figures 1A–D). Examination of FIB/SEM images revealed a good quality of fine structure and SK2 immunogold labeling and resolution comparable to that of conventional TEM (Figures 1E,F). Silver grains representing SK2 immunoreactivity were observed both at extrasynaptic sites of the dendritic membrane and at intracellular sites (Figures 1E,F). To facilitate the 3D reconstruction of dendrites immunolabelled for SK2, we first identified immunopositive dendrites of excitatory neurons (pyramidal cell dendrites with spines) and inhibitory neurons (dendrites without spines and showing numerous synaptic contacts) in a series of images (Figures 2A1–A9). Neuronal processes or individual dendrites immunopositive for SK2 present in the neuropil were clearly identifiable and could be easily followed in consecutive serial sections (Figures 2A1–A9), which is an important advantage to visualize and analyze in detail the 3D distribution. Similarly, SK2 immunoparticles on the plasma membrane and at cytoplasmic sites could be easily identified in all sections within the stack (Figures 2A1–A9).

**FIGURE 3:**
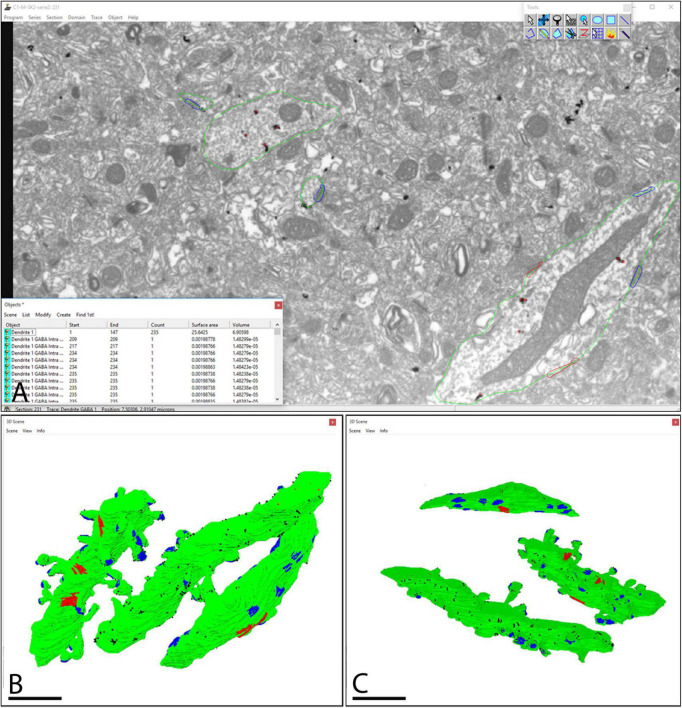
Screenshot of the software tool used for 3D reconstruction and analysis of stacks of serial sections. **(A)** The Reconstruct software interface allows visualization of the image stacks and permits the identification and 3D reconstruction of all SK2 immunoreactive neuronal elements and a list of all objects outlined to be reconstructed. Green lines outline SK2 immunoreactive dendritic shafts and spines. Immunoparticles on plasma membrane and intracellular sites are outlined in black and red, respectively. **(B,C)** The structures of interest immunoreactive for SK2 can also be represented in 3D. Two SK2 immunopositive dendrites of pyramidal cells and one of interneuron are represented and visualized in two different angles. Every reconstructed object is color-coded (surface of dendrites in green, SK2 immunoparticles in black, asymmetric synapses in blue, symmetric synapses in red), so they can be individually accessed. Scale bar: **(B,C)** 1 μm.

Following alignment of FIB/SEM images with FIJI, they were manually checked with the Reconstruct software (Figure 3A). The analysis of each individual image allowed us to trace SK2 immunoreactive neuronal elements including identified spines, their parent dendrites, and inhibitory neurons, as well as immunoparticles for SK2 along the plasma membrane and intracellular sites present on those neuron populations (Figure 3A). The identification of the same immunopositive elements in serial sections culminated with 3D reconstructions of dendritic segments (Figures 3B,C), thus facilitating the study of the molecular architecture of SK2 channels and their spatial relation to excitatory and inhibitory synapses.

### Distribution of SK2 Immunoparticles on CA1 Excitatory and Inhibitory Neurons

**FIGURE 4:**
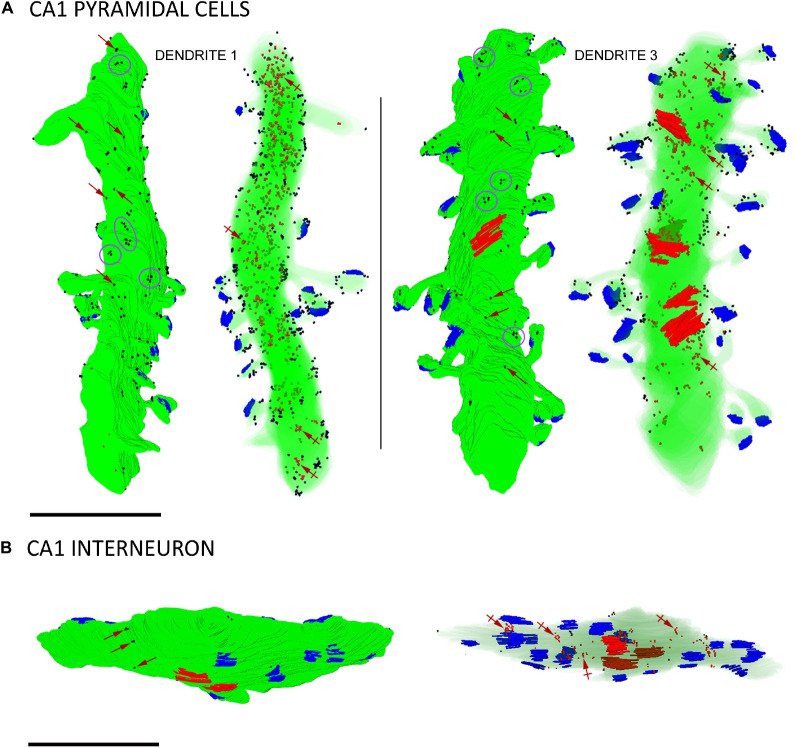
Three-dimensional reconstructions of SK2-immunopositive dendrites. **(A)** Two examples of reconstructed dendritic segments of CA1 pyramidal cells in the *stratum radiatum* immunolabelled for SK2. Dendrite 1 was reconstructed from 147 sections and dendrite 3 from 171 sections. For each dendrite, the left 3D reconstructions show SK2 immunoparticles (black dots) on the surface and the right show SK2 immunoparticles (red dots, a few indicated by crossed arrows) at intracellular sites. To visualize immunoparticles on the surface, the dendritic shaft and spines are shown in solid green. To visualize intracellular labeling the surface of dendrites are shown with transparency. Excitatory synapses on spines are shown in blue and inhibitory synapses on shafts in red. Immunoparticles for SK2 were found either isolated (red arrows) along the plasma membrane or forming clusters (purple ellipses/circles). **(B)** Example of a reconstructed dendritic segment of interneuron immunolabelled for SK2. The left and right 3D reconstructions show SK2 immunoparticles in the surface (black dots) and at intracellular sites (red dots, a few indicated by crossed arrows), respectively. Excitatory synapses on the shafts are shown in blue and inhibitory synapses in red. This dendrite was reconstructed from 88 sections and contained 18 excitatory and 3 inhibitory synapses. Few immunoparticles for SK2 were found along the plasma membrane isolated (purple arrows) along the plasma membrane, and most were distributed at intracellular sites. Scale bar: **(A,B)** 1 μm.

To date, SK2 channel distribution on pyramidal cells and its relation to glutamate release sites have been estimated from conventional immunoEM techniques (Lin et al., 2008; Ballesteros-Merino et al., 2012). Having validated the immunogold FIB-SEM technology as a reliable procedure, we reconstructed 12 dendritic segments, 6 for CA1 excitatory neurons and 6 for inhibitory neurons, seeking to precisely map the volumetric distribution of SK2 along the neuronal surface and intracellular sites (Figure 4 and Tables 1, 2). We first investigated the 3D localization of SK2 in excitatory neurons. In our samples containing 6 dendrites of excitatory neurons reconstructed (Table 1), we unambiguously identified a total number of 84 spines, all having a spine neck connecting them to the shaft and contacted by a single presynaptic axon terminal establishing an asymmetric synaptic contact. A qualitative evaluation revealed that immunoparticles for SK2 were distributed in spines and shafts following two different patterns: forming clusters, defined as 3 or more particles, and scattered or isolated, defined as only 1 or 2 particles (Figure 4A).

**TABLE 2:** Distribution of SK2 immunoparticles on 3D reconstructed dendritic shafts of interneurons. PM, Plasma membrane; Intra, intracellular.

CA1 interneurons	N° sections	N° gold	Volume (μm^3^)	Density (gold/μm^3^)	Gold PM	Gold Intra	Total
Dendrite 1	88	27	3,15	8,58	27 (12,2%)	194 (87,8%)	221
Dendrite 2	104	31	3,46	8,96	31 (12,7%)	213 (87,3%)	244
Dendrite 3	115	35	3,81	9,19	35 (13,7%)	221 (86,3%)	256
Dendrite 4	106	32	3,77	8,48	32 (12,7%)	219 (87,3%)	251
Dendrite 5	98	30	3,49	8,58	30 (11,9%)	222 (88,1%)	252
Dendrite 6	147	49	5,24	9,35	49 (14%)	301 (86%)	350
**Total**	**658**	**204**	**22,92**	**8,86 ± 0,15**	**204 (12,9%)**	**1370 (87,1%)**	**1574**

**FIGURE 5:**
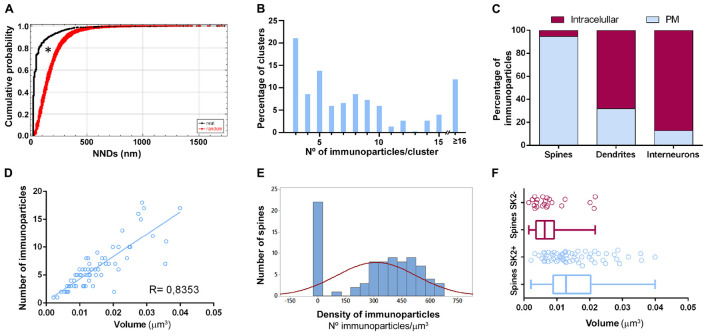
Quantitative analysis of SK2 distribution. **(A)** Cumulative probability plots of SK2-to-SK2 NND. Black and red lines show real and randomly simulated SK2, respectively. The distribution of real SK2 immunoparticles differs significantly from random distribution (*P < 0.0001). **(B)** SK2 immunoparticles composition of clusters along the neuronal surface of CA1 pyramidal cells. Sixty four percent of clusters were composed of 3–8 SK2 immunoparticles. **(C)** Percentage of SK2 immunoparticles in dendritic shafts and spines of pyramidal cells and interneurons at intracellular sites vs. plasma membrane, demonstrating differential distribution patterns. Dendritic spines contained a high proportion of SK2 along the extrasynaptic plasma membrane, while interneurons have major expression intracellularly with very low presence on the surface. **(D)** Scatter plot showing the strong positive correlation between the volume of spines and numbers of gold particles labeling SK2 (R = 0.84, Pearson’s correlation coefficient). **(E)** The histogram shows large variability in SK2 density at spines of pyramidal cells. Spines as shown in the first column (26% of total, 22 out of 84 spines) failed to display SK2 immunoparticles. **(F)** Quantitative analysis showing mean volume of spines immunopositive for SK2 (spines SK2 +) and immunonegative for SK2 (spines SK2-) in the *stratum radiatum* of the CA1 region. On average, the mean volume of spines SK2+ is significantly larger (0.0152 ± 0.001 μm^3^) than that of SK2- (0.0079 ± 0.001 μm^3^) (Mann-Whitney test, *P* < 0.001).

To demonstrate the formation of clusters, we then conducted random simulations of SK2 immunoparticles along the neuronal surface of CA1 excitatory neurons (Figure 5A). By comparing the NNDs between real distribution and one hundred simulations of the same particles on the neuronal surface, we found that the mean NND in the simulated distributions (average of 178 nm) was significantly higher than the NND in the real distribution (average of 66 nm) (Figure 5A). This indicated that immunoparticles are closer than they would be if they were randomly distributed, thus confirming that SK2 immunoparticles form clusters in the real tissue (Figure 5A). We further analyzed the immunogold composition of clusters along the neuronal surface, observing that around 64% of clusters were in the range of 3–8 immunoparticles (Figure 5B). Next, we quantitatively evaluated the relative frequency of immunoparticles on the plasma membrane and at intracellular sites of pyramidal cells (Figure 5C). This analysis revealed that from 4775 immunogold particles that were present in the dendrites reconstructed, 4360 (91%) were distributed in shafts and 415 (9%) in spines (Figure 5C and Table 1). However, spines contained a higher proportion of SK2 immunoparticles in the plasma membrane (398 out of 415; 96%) compared to dendritic shafts (1212 out of 4775; 28%) (Figure 5C and Table 1).

Immunoreactivity for SK2 was also detected in smooth dendritic shafts running through the *stratum radiatum* and identified as inhibitory neurons (Figure 4B). Therefore, we also investigated the 3D localization of SK2 in inhibitory neurons reconstructing 6 dendritic segments. In these samples, immunoparticles for SK2 were distributed along the plasma membrane in the shafts, mostly scattered or as isolated particles, as well as at intracellular sites, and not in close association with excitatory or inhibitory synapses (Figure 4B). The quantitative analysis revealed that from 1574 immunogold particles detected, 204 (12.9%) were distributed along the plasma membrane and 1370 (87.1%) at intracellular sites (Figure 5A and Table 2).

### Variability in the Density of SK2 Immunoparticles in CA1 Excitatory Neurons

All reconstructed spines were analyzed for quantitative evaluation. The number of immunoparticles labeling SK2 was variable (Table 3), but proportional to the volume of the spines (R = 0.84, Pearson’s correlation coefficient) (Figure 5D). In addition, the density of SK2 labeling varied between spines, with a mean density of immunoparticles of 312.3 ± 23.3 (Figure 5E and Table 3). A significant proportion of the reconstructed spines (22 out of 84 spines; 26%) was immunonegative for SK2 (Figure 5E). We measured the volume of these spines lacking SK2 and found that they were significantly smaller (0.0079 ± 0.001 μm^3^) than those spines immunopositive for SK2 (0.0152 ± 0.001 μm^3^) (Mann-Whitney test, *P* < 0.001) (Figure 5F).

**TABLE 3:** Number and density of immunoparticles for SK2 on 3D reconstructed dendritic spines and shafts of CA1 pyramidal cells.

CA1 pyramidal cell		Dendritic shafts			Spines			
	N° sections	N° gold	Volume (μm^3^)	Density (gold/μm^3^)	N° spines	N° gold	Volume (μm^3^)	Density (gold/μm^3^)
Dendrite 1	147	444	6,67	66,59	13	98 (1–17)	0,25	416,85 ± 30,77
Dendrite 2	115	353	3,76	93,92	11	49 (2–10)	0,14	354,45 ± 24,47
Dendrite 3	171	167	4,67	35,78	27	118 (0–18)	0,35	304,14 ± 45,46
Dendrite 4	156	48	3,74	12,82	6	14 (0–10)	0,07	119,52 ± 76,45
Dendrite 5	79	48	1,81	26,41	6	14 (0–11)	0,06	95,79 ± 60,80
Dendrite 6	155	152	4,24	35,82	21	105 (0–16)	0,26	352,94 ± 51,63
**Total**	**823**	**1212**	**23,89**	**45,22 ± 12,12**	**84**	**398 (0–18)**	**1,13**	**312,30 ± 23,26**

### Neuronal Class-Dependent Density of SK2 Channels

**FIGURE 6:**
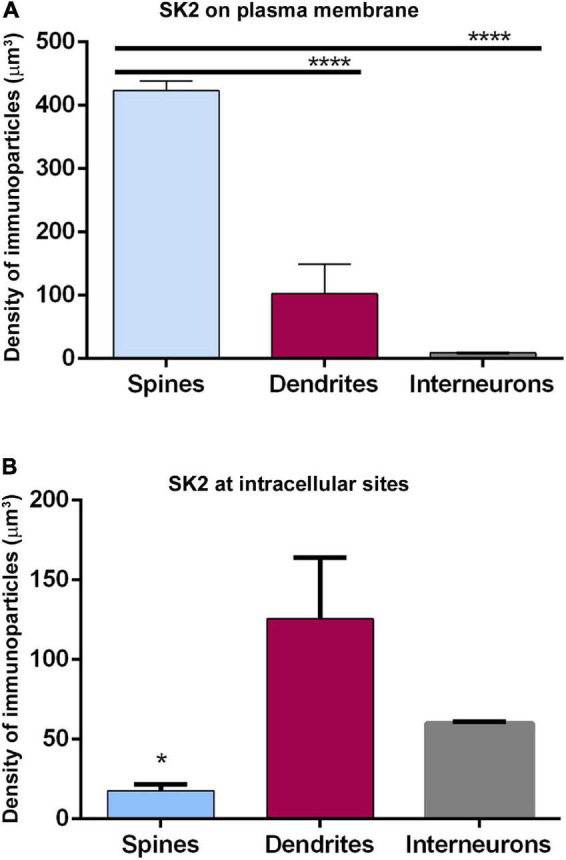
Cell type-dependent density of SK2 channels. **(A,B)** Quantitative analysis showing comparative densities, measured as immunoparticles/μm^3^ of SK2 immunoparticles along the plasma membrane (panel **A**) and intracellular sites (panel **B**) in the spines and shafts of pyramidal cells and interneurons. The density of plasma membrane SK2 on spines is 4 times higher than in shafts and 47 times higher than in interneurons. The density of intracellular SK2 immunolabelling at spines is 7-fold lower than in shafts and 3.5-fold lower than in interneurons (One-way ANOVA test and Bonferroni *post hoc* test, *p < 0.05, ****p < 0.0001). Error bars indicate SEM.

Finally, we compared the density of immunoparticles for SK2 in spines and dendritic shafts of CA1 excitatory neurons, and shafts of inhibitory neurons (Figure 6). In pyramidal cells, the data revealed that the density SK2 immunoparticles on the neuronal surface of spines (423.1 ± 15.13 immunoparticles/μm^3^; *n* = 62 spines) is 4-fold higher than the density of SK2 immunoparticles in dendritic shafts (102.4 ± 46.43 immunoparticles/μm^3^; *n* = 6 shafts) (Figure 6A and Table 3). For inhibitory neurons, the density of SK2 immunoparticles on the neuronal surface of shafts (8.9 ± 0.1 immunoparticles/μm^3^; *n* = 6 shafts) is 47-fold lower than spines and 11-fold lower than dendritic shafts of pyramidal cells (Figure 5A and Tables 2, 3). The density of intracellular SK2 immunolabelling in spines (17.5 ± 4.1 immunoparticles/μm^3^) is 7-fold lower than the density in dendritic shafts (125.6 ± 38.2 immunoparticles/μm^3^) (Figure 6B). In inhibitory neurons, the density of SK2 immunoparticles (60.0 ± 1.0 immunoparticles/μm^3^) is 3.5-fold higher than spines and 2-fold lower than dendritic shafts of excitatory neurons (Figure 6B). In summary, these findings show clear differences in the distribution of the ion channels in a neuronal class-dependent manner, as well as in pyramidal cells a subcellular compartment-dependent distribution of SK2 channels.

### Association of SK2 Channels With Excitatory Synapses on Excitatory Neurons

**FIGURE 7:**
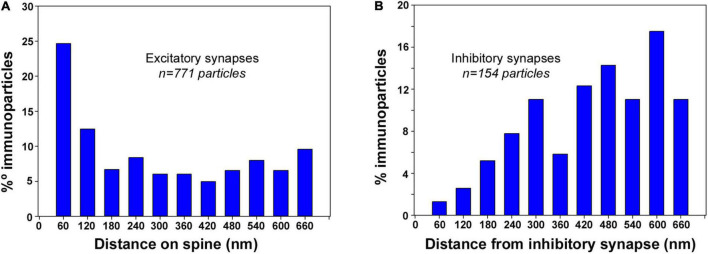
Synapse-dependent association of SK2 channels. Distribution of SK2 immunoparticles relative to the neurotransmitter release sites. Immunoparticles were recorded in 60-nm-wide bins along the extrasynaptic plasma membrane. Data are expressed as the proportion of immunoparticles at a given distance from the edge of the synaptic specialization. **(A)** Histogram showing the 3D distribution of immunoparticles for SK2 around asymmetrical synapses on spines of CA1 pyramidal cells. This data shows an enrichment of SK2 in the proximity of asymmetrical synapses on spines. **(B)** Histogram showing the 3D distribution of immunoparticles for SK2 around symmetrical synapses on dendritic shafts of CA1 pyramidal cells. This data suggests no association of SK2 with symmetrical synapses on shafts.

To gain further insight into the 3D subcellular compartmentalization of SK2 channels in CA1 pyramidal cells, we investigated the distribution of SK2 immunoparticles in relation to both glutamatergic (asymmetric) synapses in spines and in GABAergic (symmetric) synapses in dendritic shafts (Figures 7A,B). The distances of SK2 immunoparticles from the edge of asymmetric and symmetric synaptic specializations were measured in each image of the serial reconstruction. For asymmetric synapses, a total of 1631 immunoparticles were analyzed of which 771 were distributed within the first 660 nm and 860 a further distance. For symmetric synapses, a total of 610 immunoparticles were analyzed of which 154 were distributed within the first 660 nm and 456 a further distance. For this study, the limit for plotting the distribution of immunoparticles was within 660 nm.

In spines of CA1 excitatory neurons, distribution of SK2 showed a peak at perisynaptic positions, with 25% of immunoparticles being found within the first 60 nm, and 60% between 0 and 300 nm from excitatory synapses (Figure 7A). In dendritic shafts of CA1 excitatory neurons, however, only 1% of SK2 immunoparticles were distributed at perisynaptic positions, and 66% between 420 and 660 nm from inhibitory synapses (Figure 7B). This analysis revealed the enrichment of SK2 in the vicinity of glutamatergic synapses on spines but a lack of association with GABAergic synapses on dendritic shafts.

## Discussion

In the present study, we obtained volumetric estimates for the distribution of extrasynaptic SK2 channels on identified hippocampal neuron classes, excitatory neurons (CA1 pyramidal cells) and inhibitory neurons, using pre-embedding immunogold combined with FIB/SEM (immunogold FIB/SEM) and 3D quantitative analyses. Our results show that SK2 channels are preferentially located in spines of pyramidal cells and are expressed at low levels in inhibitory neurons, indicating a **neuron class**-dependency. Our data also show that the number and density of SK2 is largely variable but proportional to the volume of the spines. However, not all spines have extrasynaptic SK2 channels. The variability in SK2 channel number and density at spines, and the association of SK2 with excitatory but not inhibitory synapses in pyramidal cells, suggests differences in molecular co-assembly with Ca^2+^ sources that could influence their role in synaptic transmission and plasticity.

### Immunoreactive Signal in FIB/SEM: Advantages and Disadvantages

When performing pre-embedding immunogold techniques on different brain regions, the small size of the resulting immunoparticles requires imaging by conventional transmission electron microscopy (TEM) to identify the neuronal compartment where they are located. Although TEM of serial ultrathin sections from immunoreacted tissue is a useful approach for 3D EM (Padgett et al., 2012; Fajardo-Serrano et al., 2013; Ballesteros-Merino et al., 2014), this methodology is labor intensive, time-consuming and needs very skilful staff to obtain serial sections and TEM images, resulting in the reconstruction of small volumes. However, the renewed interest in electron microscopy during last two decades favored the development of the FIB/SEM technology, designed to capture serial electron micrographs automatically and to improve imaging speed on a larger scale [reviewed by Merchán-Pérez et al. (2009), Briggman and Bock (2012), Kubota (2015)]. When applied to the brain, this EM system allows capturing and reconstructing volumes of nervous tissue at an unprecedented scale, thus contributing to correlate structural and functional information with nanoscale resolution. In the present study, we showed that conventional pre-embedding immunogold labeling for TEM can be applied in conjunction with FIB-SEM to collect 3D ultrastructural and biomolecular information simultaneously.

Here, we clearly show that immunoparticles for SK2 appeared as dark labels in FIB/SEM images and are as easily recognizable as those in TEM images (Ballesteros-Merino et al., 2012). Our novel immunogold FIB/SEM technique proved to be highly useful in the 3D reconstruction of SK2 immunoreactive dendrites and all spines emerging from the shafts. It allowed us to quantify the number and density of SK2 immunoparticles per spine to compare the relative channel abundance in the complete dendritic arbor that had been reconstructed. On the other hand, labeling in pre-embedding approaches is performed on thick sections and reagents must penetrate the tissue through the free surfaces. Therefore, we cannot rule out the possibility that variability in the density of SK2 immunolabelling obtained for each reconstructed dendrite of pyramidal cells could be due to decreased penetration into the tissue section of the primary and/or secondary antibodies reaching dendritic segments emerging from pyramidal cells located at different distances from the surface. However, this method does show some of the disadvantages that are usually associated with the pre-embedding immunogold reactions. For instance, synaptic molecules present at the membrane specialization of glutamatergic synapses are not generally detected using this method (Luján et al., 1996). In our study, this limitation implied that we could only account for extrasynaptic SK2 channels only and not for synaptic SK2. Another common disadvantage which is associated with the pre-embedding immunogold method, i.e.,: that density of immunoparticles decreases gradually in the depth of the tissue due to the limited penetration of reagents, does not fully apply when combined with FIB/SEM due to the acquisition of 3D images with a high z-axis resolution (i.e., very thin sections) (Merchán-Pérez et al., 2009). Yet, we cannot rule out the possibility that variability in the density of SK2 immunolabelling obtained for each individual reconstructed dendrite of excitatory neurons could be due to reagents reaching dendritic segments emerging from pyramidal cells located at different depth from the surface.

### Three-Dimensional Arrangement of SK2 Channels in Hippocampal Neurons

CA1 pyramidal cells express SK2 mRNA and protein in the developing and adult hippocampus (Stocker et al., 1999; Ballesteros-Merino et al., 2012). Previous bi-dimensional electron microscopic studies on single sections have shown that SK2 channels are present in the somata of excitatory neurons, and preferentially located in their spines and shafts (Ballesteros-Merino et al., 2012; García-Negredo et al., 2014). Here, using quantitative three-dimensional approaches on serial sections, we have extended those findings showing that although SK2 can be located at any position along the somato-dendritic domain of pyramidal cells, the density of the ion channel on spines is much higher than elsewhere. In addition, the simultaneous visualization of excitatory and inhibitory synapses across the reconstructed dendrites of excitatory neurons, allowed us to demonstrate that SK2 channels are associated with excitatory synapses and glutamatergic inputs to spines, with no apparent association with inhibitory synapses from local inhibitory neurons. In agreement with this data, previous studies have demonstrated a similar synapse-dependent localization of SK2 channels in Purkinje cells (Ballesteros-Merino et al., 2014).

The preferential localization of SK2 channels in spines and excitatory synapses reflects their non-uniform distribution across the dendritic arbors. One important finding with physiological relevance, is that SK2 forms clusters *in vivo* along the neuronal surface of pyramidal cells. Previous studies have demonstrated that SK2 was susceptible to form clusters in heterologous expression systems (Strassmaier et al., 2005). Our findings of the formation of clusters of SK2 in both spines and shafts could be favored by the formation of macromolecular complexes with associated proteins like protein kinase CK2, protein phosphatase 2A (PP2A) and membrane palmitoylated protein 2 (MPP2), which could impede their diffusion in plasma membranes (Allen et al., 2007; Kim et al., 2016). In addition to those molecules regulating the activity of SK2 channels, we also reported their co-assembly forming stable complexes with mGlu_5_ receptors (García-Negredo et al., 2014). Interestingly, double-label immunogold electron microscopy using SDS-FRL revealed co-clustering of SK2 and mGlu_5_ in spines and shafts of CA1 pyramidal neurons (García-Negredo et al., 2014). This co-clustering results in functional coupling. Thus, a pharmacological stimulation of mGlu_5_ receptors mobilized intracellular Ca^2+^ to activate SK2 channels and block the activity of SK2 channels regulated mGlu_5_ receptor-mediated signal transduction (García-Negredo et al., 2014).

Inhibitory neurons present in the *stratum radiatum* form a heterogeneous population of cells (Pelkey et al., 2017; Booker and Vida, 2018). Although the morphology and synaptic inputs of these inhibitory neurons are well characterized (Booker and Vida, 2018), reports of the subcellular localization of SK2 on inhibitory neurons located in this and other regions of the CA1 field are still lacking. SK2 transcripts have been described in the *stratum radiatum* using *in situ* hybridization (Stocker et al., 1999). Consistent with these data, our results showed that SK2 is present on the surface of inhibitory neurons, although at a low density, and more frequently at intracellular sites. Inhibitory neurons immunolabelled for SK2 could not be identified as any specific subtype in the absence of information about their axonal patterns or neurochemical characteristics, but they probably represent the same population of mGlu_5_ immunolabelled inhibitory neurons (Luján et al., 1996).

### Large Variability in the Density of SK2 Channels in the Plasma Membrane

One of the most significant findings of the present study is that spines of excitatory neurons form a heterogeneous population in relation to the SK2 channel content. We report here that the degree of immunolabelling for SK2 channels correlates strongly with the size of spines, thus the larger the spine, the larger the density of SK2 channels. In addition, our 3D reconstructions provide direct evidence for the presence of a significant population of spines lacking extrasynaptic SK2 channels and that these are smaller in size to SK2 immunopositive spines. Interestingly, morphology and size of spines is linked to synaptic efficacy and long-term potentiation of synaptic strength. Small spines seem to be specialized for plasticity, while large spines have been proposed to serve as “memory” neuronal structures (Kasai et al., 2003; Bourne and Harris, 2007). Together with our data, this suggests that SK2 channels may subserve specific roles in different spines.

Our data on the segregation of extrasynaptic SK2 channels to different populations of spines, do not exclude the possibility that synaptic SK2 channels are still present in PSDs (Lin et al., 2008). Furthermore, this has been supported by functional experiments performed in CA1 pyramidal cells suggesting that separate populations of SK2 channels occupy discrete subcellular positions where they couple with different Ca^2+^ sources to serve distinct physiological roles (Ngo-Anh et al., 2005). High-resolution immunogold techniques demonstrated such a segregation within spines, with a molecular and functional association of SK2 with NMDA receptors at PSDs (Lin et al., 2008) and SK2 with mGlu_5_ receptors at extrasynaptic sites (García-Negredo et al., 2014).

We can speculate that the differential expression of extrasynaptic SK2 channels may reflect differences in the localization of SK2 isoforms in spines. The *Sk2* gene directs expression of two isoforms, SK2 *long* and SK2 *short*, both differing in the extra 207 amino acids at the N terminus of SK2 long and is expressed in many of the same brain regions (Strassmaier et al., 2005). Immunoelectron microscopy using SDS-FRL has demonstrated that channels containing SK2 *short* were located on the extrasynaptic plasma membrane of spines, but they were selectively excluded from the PSD (Allen et al., 2011a). Further experiments using double labeling immunogold are necessary to further probe this molecular segregation.

In summary, to our knowledge, this study is the first to combine a pre-embedding immunogold technique for an ion channel with FIB/SEM to reveal volumetric organization in hippocampal neurons. This new approach offers a new tool to gain functional and structural information with 3D nanoscale resolution. Nearest distance measurements between immunoparticles and distance to synaptic specializations, in 3D space, can also be performed providing many capabilities to comprehensively analyze many aspects of SK2 organization in pyramidal cells. The immunogold FIB/SEM proved to be a powerful tool to analyze tri-dimensionally local neural circuits at the subcellular level in the brain.

## Data Availability Statement

The raw data supporting the conclusions of this article will be made available by the authors, without undue reservation.

## Ethics Statement

The animal study was reviewed and approved by Comité Ético de Experimentaci n Animal (CEEA) of UCLM.

## Author Contributions

RL and JD designed the project. AM-P performed FIB/SEM. JS performed 3D quantitative analysis. AM-B, RA-R, CA, and AM-M performed pre-embedding immunoelectron microscopy and quantitative analysis. RL, AM-B, and RA-R performed 3D reconstructions. RL wrote the manuscript. All authors had full access to all data in the study and take responsibility for the integrity of the data, the accuracy of the data analysis, read and approved the final manuscript.

## Conflict of Interest

The authors declare that the research was conducted in the absence of any commercial or financial relationships that could be construed as a potential conflict of interest.

## Publisher’s Note

All claims expressed in this article are solely those of the authors and do not necessarily represent those of their affiliated organizations, or those of the publisher, the editors and the reviewers. Any product that may be evaluated in this article, or claim that may be made by its manufacturer, is not guaranteed or endorsed by the publisher.

## Footnotes

1
http://fiji.sc
